# Bone Marrow Defects and Platelet Function: A Focus on MDS and CLL

**DOI:** 10.3390/cancers10050147

**Published:** 2018-05-18

**Authors:** Sarah Luu, Elizabeth E. Gardiner, Robert K. Andrews

**Affiliations:** 1 Australian Centre for Blood Diseases, Monash University, Melbourne, VIC 3004, Australia; sarah.luu@monash.edu; 2 ACRF Department of Cancer Biology and Therapeutics, The John Curtin School of Medical Research, The Australian National University, Canberra, ACT 2600, Australia; elizabeth.gardiner@anu.edu.au

**Keywords:** platelets, bone marrow defects, glycoprotein Ibα, glycoprotein VI

## Abstract

The bloodstream typically contains >500 billion anucleate circulating platelets, derived from megakaryocytes in the bone marrow. This review will focus on two interesting aspects of bone marrow dysfunction and how this impacts on the quality of circulating platelets. In this regard, although megakaryocytes are from the myeloid lineage leading to granulocytes (including neutrophils), erythrocytes, and megakaryocytes/platelets, recent evidence has shown that defects in the lymphoid lineage leading to B cells, T cells, and natural killer (NK) cells also result in abnormal circulating platelets. Current evidence is limited regarding whether this latter phenomenon might potentially arise from (a) some form of as-yet-undetected defect common to both lineages; (b) adverse interactions occurring between cells of different lineages within the bone marrow environment; and/or (c) unknown disease-related factor(s) affecting circulating platelet receptor expression/function after their release from megakaryocytes. Understanding the mechanisms underlying how both myeloid and lymphoid lineage bone marrow defects lead to dysfunction of circulating platelets is significant because of the potential diagnostic and predictive value of peripheral platelet analysis for bone marrow disease progression, the additional potential effects of new anti-cancer drugs on platelet function, and the critical role platelets play in regulation of bleeding risk, inflammation, and innate immunity.

## 1. Introduction

**Figure 1:**
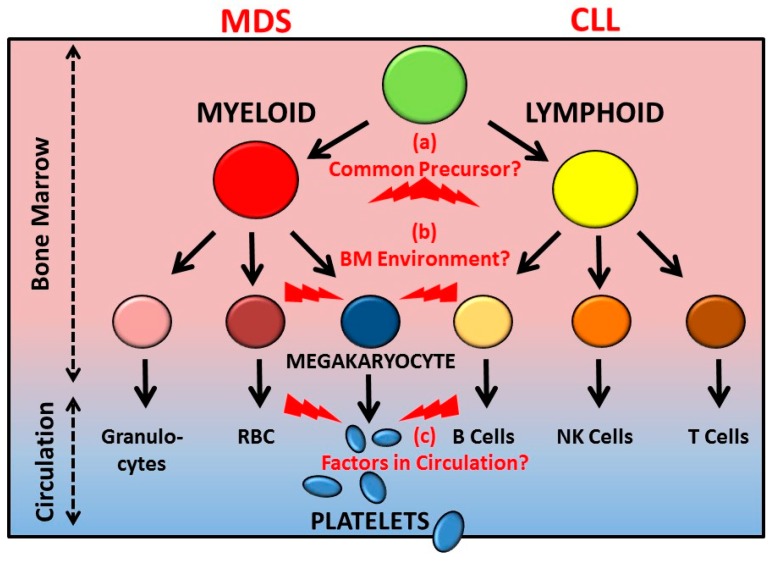
Haematopoietic cells are derived from the myeloid lineage leading to generation of granulocytes (including neutrophils), erythrocytes (red blood cells; RBC), and megakaryocytes that generate platelets, or from the lymphoid lineage producing immune B cells, T cells, and natural killer (NK) cells. Defects in the lymphoid lineage pathways surprisingly also lead to abnormal circulating platelets. Dysfunction of circulating platelets may be associated with myeloid lineage bone marrow defects such as myelodysplastic syndrome (MDS) or lymphoid lineage defects such as chronic lymphocytic leukemia (CLL), by unknown mechanisms potentially involving (a) some form of defect common to both lineages; (b) adverse interactions occurring between cells of different lineages within the bone marrow environment; and/or (c) unknown disease-related factor(s) affecting circulating platelet receptor expression/function after their release from megakaryocytes. Understanding mechanisms may be significant for understanding increased risks of bleeding or infection. See the text for references.

The bloodstream typically contains >500 billion circulating anucleate platelets (~150–400 × 10^9^/L in ~4–5 L of blood) derived from megakaryocytes primarily situated in the bone marrow. In simple terms, megakaryocytes are within the myeloid lineage leading to granulocytes (including neutrophils), erythrocytes, and platelets, whereas the lymphoid lineage, derived from a common precursor, leads to B cells, T cells, and natural killer (NK) cells (Figure 1). Together, these different types of cells released into the vasculature play a crucial role in the transfer of oxygen, nutrients, and waste products throughout the body, in preventing blood loss following injury and promoting wound healing, and in fighting infection through innate and adaptive immune defense strategies [1,2,3]. On the other hand, dysfunction or deficiency of one or more of these cells due to bone marrow defects or other causes can result in bleeding, thrombotic disease, coagulopathy, inflammatory disease, increased infection risk, and/or (auto)immune disorders, with various cells typically playing multiple roles in initiating or regulating these interrelated health/disease processes.

In this review, we will briefly highlight the role of human blood platelets in haemostasis and thrombosis, procoagulant and pro-inflammatory responses, innate immunity, and cancer. In this respect, although individuals may be thrombocytopenic, with platelet count <150 × 10^9^/L, in the absence of other causes, bleeding is generally not a major risk until platelets are <5–10 × 10^9^/L, suggesting there are far greater numbers of platelets in the circulation than required for their primary haemostatic function, and consistent with their expanding roles in other areas of vascular biology. In the absence of thrombocytopenia, platelets may be dysfunctional due to a plethora of causes, either inherited or acquired. This review will then consider how bone marrow defects of the myeloid or lymphoid lineage may result in defects of circulating platelets, using myelodysplastic syndrome (MDS) and chronic lymphocytic leukemia (CLL), respectively, as cases in point.

## 2. Platelet Function and Key Receptors, Including Roles in Cancer/Metastasis

In haemostasis, circulating platelets in flowing blood rapidly adhere at sites of vascular injury, become activated, secrete platelet agonists, procoagulant and pro-inflammatory factors from storage granules, release microparticles, and activate αIIbβ3 and other integrins that mediate platelet aggregation or thrombus formation. The activated platelet surface through exposure of phosphatidyl serine and other pathways promotes the generation of thrombin, a potent human platelet agonist that engages protease-activated receptors (PAR)-1 and 4, and thrombin-dependent fibrin formation in coagulation. The key platelet-specific receptors glycoprotein (GP)Ibα of the GPIb-IX-V complex and GPVI are critical for initiating platelet adhesion and activation particularly at elevated shear rates in flowing blood [4,5]. GPIbα of the leucine-rich repeat protein family binds von Willebrand factor (VWF), is a high-affinity binding site for thrombin, and also binds other coagulation factors (XII, XI, high molecular weight kininogen) and receptors P-selectin expressed on activated platelets or activated endothelial cells and αMβ2 on activated neutrophils; P-selectin expressed on the surface of activated platelets also adheres to P-selectin glycoprotein ligand-1 (PSGL-1) on neutrophils [6]. GPIbα forms a disulfide-linked complex with GPIbβ, and noncovalently associates with GPIX and GPV, all members of the leucine-rich repeat family homologous to mammalian innate immune toll-like receptor (TLR) proteins [7] and to primitive defensive proteins (variable lymphocyte receptors) expressed in the jawless vertebrates, lampreys and hagfish [8]. GPV is involved in adhesion of the complex to collagen [9]. In addition, GPIbα also forms a noncovalent complex with GPVI on the human platelet surface [10]. GPVI of the immunoglobulin-like family is co-associated with the Fc receptor (FcR)γ, required for GPVI surface expression. FcRγ contains an immunoreceptor tyrosine-based activation motif (ITAM) linking engagement of GPVI/FcRγ by physiological ligands collagen and fibrin or non-physiological ligands such as cross-linked collagen-related peptide (CRP) or convulxin, to Src family kinase signalling pathways [4,5]. Conserved cytoplasmic sequences of GPIb-IX-V/GPVI are directly linked to various intracellular signalling/cytoskeletal proteins, including tumour necrosis factor (TNF) receptor-associated factor-4 (TRAF-4)/p47*^phox^* of the nicotinamide adenine dinucleotide phosphate (NADPH) oxidase (Nox) complexes generating intracellular reactive oxygen species (ROS) following engagement of GPIbα or GPVI [11,12], and the p85 subunit of phosphatidyl inositol-3 (PI-3) kinase [13,14]. The major signalling pathways downstream of both GPIb-IX-V and GPVI engagement include Src, PI3-kinase, and spleen tyrosine kinase (Syk), leading to activation of phospholipase Cγ (PLCγ) and mobilization of intracellular Ca^2+^, and platelet aggregation in response to selective engagement of GPIbα is blocked by inhibitors of Src, PI3-kinase, and Syk [15]. GPVI forms dimers on the platelet surface, and ligand-induced cross-linking or clustering of GPVI/FcRγ also leads to rapid Src kinase-dependent ITAM-mediated Syk phosphorylation, and activation of PLCγ leading to Ca^2+^ mobilization [16,17,18,19].

Importantly, cytoplasmic sequences of GPIb-IX-V/GPVI are also directly linked to calmodulin [20,21,22] which regulates a disintegrin and metalloproteinase (ADAM)-dependent extracellular shedding of ligand-binding domains of GPIbα (ADAM17), GPV (ADAM10/17), and GPVI (ADAM10) [23]. Plasma soluble GPVI (sGPVI) as well as soluble GPIbα and GPV, constitute unique platelet-specific biomarkers of platelet activation or dysfunction, with sGPVI levels in particular linked to specific bleeding risk [24,25]. Such regulation of platelet surface receptor density is likely to be increasingly studied with respect to adhesive function, signalling functions, interaction with endothelial cells or leukocytes, and clearance of platelets by macrophage adhesion in the liver [26,27]. Compared to existing antiplatelet drugs [28,29,30], all of which are limited to some extent by low efficacy or high bleeding risk, new targets including GPIbα and GPVI are emerging and antiplatelet therapy may have wider application in other human disease.

During inflammation, platelets adhere to neutrophils via GPIbα/αMβ2 and P-selectin/PSGL-1 interactions, and regulate angiogenesis and vascular integrity via GPVI, αIIbβ3 and the platelet C-type lectin-like receptor 2 (CLEC-2) [30,31,32,33]. Platelets also interact with bacteria and other pathogens via GPIbα, GPVI, αIIbβ3, and other mechanisms [34,35,36]. In addition to pro-inflammatory cytokines, activated platelets also release growth factors and microparticles which could all potentially play an important role in cancer, albeit the association between abnormal platelet function related to bleeding or thrombotic/coagulopathy risk, immunosuppression and cancer survival, growth and/or metastasis is complex [37,38,39,40,41,42,43,44,45,46,47,48,49,50,51]. Platelets may interact with metastatic cancer cells entering the blood stream via receptor-mediated adhesion and/or release of soluble factors, contribute to suppression of immune response towards cancer cells [52], and/or regulate localization within the microvasculature to enable redistribution and enhance survival. Coagulopathy, including contact or tissue factor pathways, activated platelets, microparticles, thrombin generation, and platelet thrombin PAR receptors, may also play a role in cancer-related thrombosis [53,54,55]. The cause or effect of changes in platelet profiles and cancer is not always clear [46], however platelets may ultimately offer new anti-tumour therapeutic approaches [38,48,49,50,51,56,57]. Circulating platelets could therefore be informative regarding mechanisms, constitute therapeutic targets, and/or be useful diagnostically on progression or response to treatment. In this regard, platelet RNA is also emerging as an interesting new diagnostic parameter [58,59]. Here, we will consider two examples where acquired changes in platelet receptor expression or function have been reported, in myeloid or lymphoid blood cancers.

## 3. Myelodysplastic Syndrome (MDS) and Platelet Function

MDS is characterized by ineffective production (dysplasia) of myeloid blood cells (Figure 1), with accompanying risk of transformation to acute myelogenous leukaemia (AML). The underlying causes of MDS affect adults and less frequently children. These causes are heterogeneous, with multiple clonal haematopoietic defects identified and including germ line inheritable forms, in addition to a number of predisposing factors, and currently the main therapy involves haematopoietic stem cell transplantation [60,61]. There may be a variable extent of thrombocytopenia and other platelet/haemostatic defects associated with MDS, and there may be bleeding episodes of varying severity.

An acquired platelet GPVI defect associated with MDS has been reported, with essentially normal platelet counts and GPVI expression, but with no aggregation to platelet GPVI ligands (collagen, CRP, or convulxin), and with ultimate progression to AML [62]. Subsequent studies of 26 patients with MDS also showed platelet-related defects unrelated to thrombocytopenia, including high prevalence (>80%) of defective aggregation in response to collagen or other agonists (adrenaline, adenosine diphosphate (ADP), ristocetin) [63]. Defective platelet aggregation also strongly correlated with poor prognosis, although the underlying mechanism is not currently known. A further case of confirmed MDS-low risk with normal platelet count and increased bleeding tendency (recurring episodes over the previous 18 months), also showed defective GPVI-dependent aggregation (collagen, CRP), abnormally low GPVI surface expression on platelets and a signalling defect associated as a molecular abnormality in proteolytic processing of phosphorylated Syk [64]. Further, it was found that GPVI ligation induced intracellular ROS generation, independent of Syk activation or pathways leading to platelet aggregation or ectodomain shedding. There was a gradual decline in platelet count over several months, and also progression to AML [64]. Another study of 75 MDS cases with varying degrees of thrombocytopenia showed defective platelet activation, as well as reduced circulating immature platelet fraction and an increase of apoptotic markers in platelets consistent with defective platelet production [65].

While platelet GPVI-related dysfunction is associated with a significant proportion of MDS cases, further studies are no doubt required to fully evaluate these changes temporally and in response to treatment in order to discover the molecular link between specific defects and platelet function, and to establish any prognostic value in future. While the underlying mechanisms could speculatively involve either direct or indirect targeting of particular signalling or cytoskeletal proteins involved in GPVI expression or function, it is clear that more detailed experimental analysis perhaps involving megakaryocyte development and platelet production together with advanced screening of MDS is required to fully understand the precise causes and effects related to the altered platelet phenotype.

## 4. Chronic Lymphocytic Leukemia (CLL) and Platelet Function

CLL is characterized by defects in development of B-lymphocytes (Figure 1), but can also be associated with platelet/haemostatic dysfunction [66,67,68,69]. Ibrutinib, one of a number of recent drugs developed for the treatment of CLL, targets Bruton’s tyrosine kinase (Btk), a signalling molecule that plays an essential role in B-cell development [66,69,70]. While reasonably well tolerated in clinical trials, its expanding use has corresponded with an increased bleeding risk, as well as other cardiovascular complications and infections [66]. Ibrutinib treatment may also result in off-target inhibition of additional Src family kinase members [71]. Btk has previously been linked to signalling pathways downstream of platelet GPIbα and regulation of GPIbα-dependent thrombus formation [72], consistent with subsequent studies reporting an inhibitory effect of Ibrutinib on GPIbα/GPVI-dependent platelet function and potential exacerbation of bleeding risk [73,74,75]. Interestingly, circulating platelets from a small cohort of untreated refractory CLL patients showed significantly decreased surface expression of GPIbα and GPVI, whereas surface expression of αIIbβ3 and CD9 (control) were essentially normal [76]. Together, this suggests that Ibrutinib or other signalling pathway inhibitors [70] could exacerbate bleeding disorders in platelets already compromised by abnormally low receptor expression [76]. That is, while evidence for platelet dysfunction associated with CLL is more limited than is the case for MDS discussed above, and while spontaneous or clinically significant bleeding is not typically reported as being associated with CLL, significantly decreased levels of expression of GPIbα and GPVI, together with mildly reduced platelet count and/or the increased susceptibility to bleeding in the presence of anti-cancer drugs that impact upon key platelet signalling pathways, may ultimately have consequences in terms of monitoring bleeding risk or use of antiplatelet agents. Also, given the emerging non-haemostatic role for platelets mentioned above [1,2,3,30,31,32,33,34,35,36], defects in expression of GPIbα/GPVI could potentially be related to changes in inflammatory/infection risk or immune dysfunction. These platelet abnormalities prior to treatment could also be relevant to bleeding associated with use of other CLL drugs, such as those targeting B cell CLL/lymphoma 2 (BCL-2) proteins that regulate apoptosis/survival. Whereas a subsequent BCL-2-selective drug (ABT-199) had less impact on platelet function [77], pan-BCL-2 inhibitory drugs that target both BCL-2 and BCL-2-like protein 1 (ABT-737, ABT-263) resulted in pronounced transient thrombocytopathy and functional impairment of αIIbβ3 together with enhanced shedding of GPIbα and GPVI, resulting in markedly defective thrombus formation [78]. Further, unlike immune-related thrombocytopenia where anti-platelet antibodies acting at the FcγRIIa receptor markedly increase GPVI shedding resulting in lower surface expression and increased levels of sGPVI in plasma [76,79,80], in untreated CLL the plasma sGPVI levels were in the normal range, suggesting observed low platelet GPVI was unlikely to be the result of increased shedding.

Finally, current evidence is limited regarding whether the effect of CLL-related defects on circulating platelet profile might potentially result from (a) some form of as-yet-undetected defect common to both lineages; (b) adverse interactions occurring between cells of different lineages within the bone marrow environment; and/or (c) unknown disease-related factor(s) affecting circulating platelet receptor expression/function after their release from megakaryocytes. As CLL is not associated with either a unique cytogenetic or a molecular defect, mouse models of CLL generally recapitulate only some aspects of the disease, however it would be of interest to examine platelet and megakaryocyte function in one or more mouse models of progressive CLL [81]. As for MDS discussed above, further studies are now required to evaluate temporal changes in platelet receptor expression in early and late stage CLL, in the absence or presence of drugs or other treatment(s), to determine the relationship to platelet function and bleeding risk, as well as to establish any future clinical value in such analysis.

## 5. Conclusions and Future Directions

The heterogeneous nature of MDS and CLL is unlikely to result in a simple explanation for changes in platelet receptor expression and function associated with both diseases (Figure 1). In both cases, a platelet production defect could explain variation in platelet count as well as altered receptor density, although changes in circulating red blood cells, neutrophils or B cells could conceivably affect platelet receptor expression by increased platelet activation status, enhanced receptor shedding or other mechanisms. Cell co-culture experiments involving platelet production in vitro in the presence or absence of other mutant bone marrow cells could provide some useful information on the potential mechanisms involved. More extensive temporal analyses in human MDS or CLL, or the corresponding animal models, could also be informative. Finally, the extensive genetic defects underlying MDS or CLL will ultimately be interpretable as genetic abnormalities associated with congenital and acquired platelet defects are increasingly understood. In any case, these studies of platelet-specific receptors highlight the scientific and potential clinical interest in such changes in human disease. 

In conclusion, understanding the mechanisms underlying how both myeloid and lymphoid lineage bone marrow defects lead to dysfunction of circulating platelets is significant because of the potential diagnostic and predictive value of peripheral platelet analysis for bone marrow disease progression, the additional potential effects of new anti-cancer drugs on platelet function, and the critical role platelets play in regulation of bleeding risk, inflammation, and innate immunity. These findings ultimately may also be relevant to increased understanding of other human diseases (e.g., congenital platelet disorders, cardiovascular disease, stroke, autoimmune thrombocytopenia) and/or surgical procedures (e.g., circulatory support devices, splenectomy) where expression/function of platelet-specific receptors GPIbα/GPVI is compromised and there is concomitant altered haemostatic, thrombotic, inflammatory, and/or immune risk.
